# Deficiency of Adipose Triglyceride Lipase Induces Metabolic Syndrome and Cardiomyopathy in Zebrafish

**DOI:** 10.3390/ijms24010117

**Published:** 2022-12-21

**Authors:** Hsin-Hung Lai, Kun-Yun Yeh, Hung-Ming Hsu, Guor Mour Her

**Affiliations:** 1 Institute of Biopharmaceutical Sciences, National Yang Ming Chiao Tung University, Taipei 112, Taiwan; 2 Division of Hemato-Oncology, Department of Internal Medicine, Chang Gung Memorial Hospital, Keelung 204, Taiwan

**Keywords:** lipid metabolism, lipolysis, metabolic syndrome, cardiomyopathy

## Abstract

Lipid metabolism dysfunction is related to clinical disorders including obesity, cancer, liver steatosis, and cardiomyopathy. Impaired lipolytic enzymes result in altered release of free fatty acids. The dramatic change in dyslipidemia is important in lipotoxic cardiomyopathy. Adipose triglyceride lipase (ATGL) catalyzes the lipolysis of triacylglycerol to reduce intramyocardial triglyceride levels in the heart and improve myocardial function. We examined the role of ATGL in metabolic cardiomyopathy by developing an Atgl knockout (ALKO) zebrafish model of metabolic cardiomyopathy disease by continuously expressing CRISPR/Cas9 protein and *atgl* gene guide RNAs (gRNAs). The expressed Cas9 protein bound to four gRNAs targeting the *atgl* gene locus, facilitating systemic gene KO. Ablation of Atgl interfered with lipid metabolism, which induced hyperlipidemia and hyperglycemia. ALKO adults and embryos displayed hypertrophic hearts. ALKO presented a typical dilated cardiomyopathy profile with a remarkable reduction in four sarcomere genes (*myosin heavy chain 7-like***,** *actin alpha cardiac muscle 1b***,** *myosin binding protein C3*, and *troponin T type 2a*) and two Ca^2+^ handling regulator genes (*tropomyosin 4b* and *ATPase sarcoplasmic/endoplasmic reticulum Ca^2+^ transporting 2b*). Immune cell infiltration in cardiac tissue of ALKO provided direct evidence of advanced metabolic cardiomyopathy. The presently described model could become a powerful tool to clarify the underlying mechanism between metabolic disorders and cardiomyopathies.

## 1. Introduction

The metabolic complications of early obesity have become a global public health crisis. Obesity is directly related to many metabolic disorders, including metabolic syndrome, type 2 diabetes, hypertension, dyslipidemia, and cardiovascular disease (CVD) [1,2,3]. Subcutaneous or visceral fat distribution in obese patients is correlated with disease progression [4]. The deposition of adipose tissue in the epicardium has been associated with cardiomyopathy [5]. An abnormal extent of metabolite-induced inflammation may cause metabolic disorders in several tissues, including adipose [6], liver [7], vascular tissue [8], and heart [9,10].

Adipose triglyceride lipase (ATGL), also known as patatin-like phospholipase domain containing 2 (PNPLA2), dictates the lipolytic process of triglycerides. It is highly expressed in adipose tissue and less defined in skeletal muscle, cardiac tissue, and other tissues [11,12]. Lipolysis of triacylglycerol is suppressed and stored in fat tissue when ATGL expression is low [13]. Low ATGL expression impairs the metabolic microenvironment, increasing the chances of obesity and hyperlipidemia [14,15]. ATGL also regulates mitochondrial function by mediating the expression of peroxisome proliferator-activated receptors, and expression of the downstream molecule peroxisome proliferator-activated receptor gamma cofactor 1 (PGC1) leads to a dramatic accumulation of lipids in myocardial tissue [16,17]. Depletion of ATGL causes a critical role in cardiac hypertrophy in mice [12,18]. Patients with ATGL mutations usually develop severe cardiomyopathy or detrimental lipid [19,20,21]. However, pharmaceutical treatments are limited because of the limited knowledge of metabolic syndrome and lipotoxic cardiomyopathy.

Zebrafish are a suitable model for human CVD via chemical induction [22,23,24] or genetic manipulation [25,26,27]. For example, Gu et al. developed a zebrafish larvae model of dilated cardiomyopathy (DCM) induced by rapid terfenadine treatment [28]. Ma et al. reported a doxorubicin-induced CM model in adult zebrafish [24]. Zhang et al. generated mto1knock-out (mto1KO) zebrafish by genome editing using the CRISPR/Cas9 system. Cardiac defects in mto1KO zebrafish recapitulated the clinical phenotypes of hypertrophic CM [29]. Genetically modified zebrafish carrying the human truncating variant of the giant sarcomeric protein titin (TTNtv) spontaneously develop DCM [30]. Lipogenic pathways [31,32] and inflammatory signaling [33,34] are also highly consistent in zebrafish and humans. The link between the underlying mechanisms of lipid metabolism and heart remodeling remains unclear. Embryonic and adult zebrafish models are advantageous in studying the initial acute and later chronic stages of cardiomyopathy to clarify novel signaling mechanisms and therapeutic strategies for CM.

Zebrafish DCM models enable a new avenue of research, including identifying cardiomyopathy modifiers via forward genetic screening. In the present study, we generated an *atgl* KO zebrafish (ALKO) using gene editing achieved by the CRISPR/Cas9 system with multiple guide RNAs (gRNAs) targeting strategies. Phenotypic traits were characterized thoroughly and the phenotypic characteristics that identified DCM in adult zebrafish were identified. The findings demonstrate that defective Atgl leads to inhibition of the lipolytic process, an activated lipogenic pathway, and a microenvironment characterized by an energy imbalance in the ALKO model. Inherited DCM is evident in the in vivo model.

## 2. Results

### 2.1. Generation of Atgl Multiple Depletions Lines in Zebrafish 

**Figure 1:**
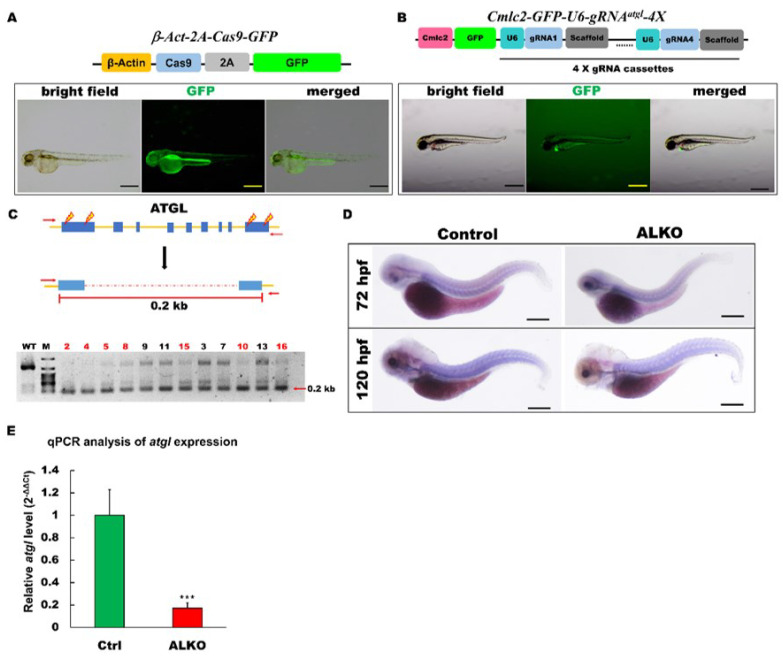
Generation of zebrafish ALKO mutant using CRIPRC-Cas9 system. (**A**) Schematics of the structure of *β-Act-2A-Cas9-GFP* expression vector. The transcriptional activity of Cas9 elements (sky blue) was driven by β-actin 2.5k promoter (yellow). The lower panel shows the fluorescence pattern of Tg (*β-Act-2A-Cas9-GFP*) with a global expression of GFP at 2 days post-fertilization (dpf). Scale bar: 500 μm. (**B**) Schematics of the structure of *Cmlc2-GFP-U6-gRNA^atgl^-4X* expression vector. The GFP expression represented the success of transgenesis of Tg (*Cmlc2-GFP-U6-gRNA^atgl^-4X)* at 5 dpf. Scale bar: 600 μm. (**C**) Upper: Schematic diagram of the outer primers of *atgl* loci used for PCR detection of mutations. The outer primers are to the mutated site/scheme of the locations of PCR primers (red arrows) designed to detect a disruption in the spacer between the first exon and the last one. Yellow lightening symbols denote the site of four selective gRNAs; navy blue boxes denote all of the exons in the *atgl* loci; red dashed line denotes the spacer; red line denotes the length between external primers without the spacer. Lower: Result of semi-qRT–PCR analyses on the whole embryo of transient F0 fish containing the corresponding mutations (as indicated in the upper panel of C). (**D**) Whole-mount in situ hybridization showing the reduction of *atgl* in ALKO mutant larvae at 120 hpf. Scale bars = 80 μm. (**E**) Quantitative of *atgl* expression by RT-qPCR analysis at 7 dpf. Heterozygous ALKO mutants were generated by cross-homozygous Tg (*β-Act-2A-Cas9-GFP*) and Tg (*Cmlc2-GFP-U6-gRNA^atgl^-4X).* Control: Heterozygous Tg (*Cmlc2-GFP-U6-gRNA^atgl^-4X*). *** (*p* < 0.001) indicates statistically significant differences from the controls. Value of *atgl* in ALKO = 0.1721 ± 0.0421.

The 4X-Cas9 genome editing method was used to establish the *atgl* mutant allele in zebrafish. This strategy aims to disrupt the target gene locus by scattering multiple gRNAs (four gRNA cassettes were applied in this study) anchored from the transcriptional start point (first exon) to the transcription endpoint (last exon). Previous studies have inspired this strategy using specific promoter-driven Cas9 and U6 promoter-driven gRNAs [35,36]. In the present study, we constructed two transgenic expression vectors (Figure 1A) to generate Tg (*βAct-2A-Cas9-green fluorescent protein [GFP]*) and Tg (*Cmlc2-GFP-U6-gRNA^atgl^-4X*). Both were maintained as F2 homozygous and crossed together to generate the 4X-*atgl* knockout (ALKO) for the following experiments.

#### 2.1.1. Generation of Transgenic Global Cas9 Expression Lines in Zebrafish

Global CRISPR/Cas9 protein expression was driven by the β-actin promoter along with GFP under the transgenic conditions (Figure 1A, upper panel). GFP served as a linear tracer of transgenic Cas9 protein expression and autocleavage by the 2A element at the translational level. The successful establishment of Tg (*β-Act-2A-Cas9-GFP*) was indicated by the global and continuous expression of GFP (Figure 1A, lower panel).

#### 2.1.2. Generation of 4X-gRNA^atgl^ Transgenic Line in Zebrafish

In contrast to other studies, we used 4X-gRNAs to target the *atgl* gene locus with four constructed gRNA cassettes. Each cassette was driven by the U6 RNA polymerase III promoter. To validate the success of the transgene, GFP expression was driven by the *cardiac myosin light chain 2* (*cmlc2*) cardiomyocyte-specific promoter [37] (Figure 1B, upper panel). After we generated the 4X-gRNA*^atgl^* transgenic zebrafish following the general Tol2 transgenic protocol, GFP was strongly expressed in the cardiac tissue of the 4X-gRNA*^atgl^* transgene (Figure 1B, lower panel).

#### 2.1.3. Combination of Global Cas9 and 4X-gRNA^atgl^ Compound Transgenic Line and Successfully Disrupted Atgl Locus in Zebrafish 

After we generated the global Cas9 and 4X-gRNA*^atgl^* transgenic strains, these two strains were crossed to impair the *atgl* gene locus in zebrafish. Insertion/deletion (indel) analysis [38,39] was performed on offspring of ALKO using external primers (forward primer: TGACTTTACCCAAAGATTATCATC; reverse primer: TCAGCCTCCTCGCATTGCGTTCTG) at five days post-fertilization (dpf). The highly relative singles of 0.2 kb size PCR products (Figure 1C, upper panel) in seven of 12 ALKO embryos compared to the wild type indicated that large-scale impairment occurred in the ALKO group by semi-quantitative RT-PCR (Figure 1C, lower panel). To prove the success of *atgl* gene editing, we measured the transcription levels of *atgl* using in situ hybridization and RT-qPCR. The in situ hybridization assay revealed a significant reduction in *atgl* transcripts in ALKO embryos, 72 h post-fertilization (hpf), or 120 hpf (Figure 1D). Quantitative expression of *atgl* in ALKO was also detected in the RT-qPCR analysis, with a dramatic five times decrease evidently compared to the control group (Figure 1E).

### 2.2. Depletion of Atgl Lead a Metabolic Imbalance in Zebrafish Larvae

**Figure 2:**
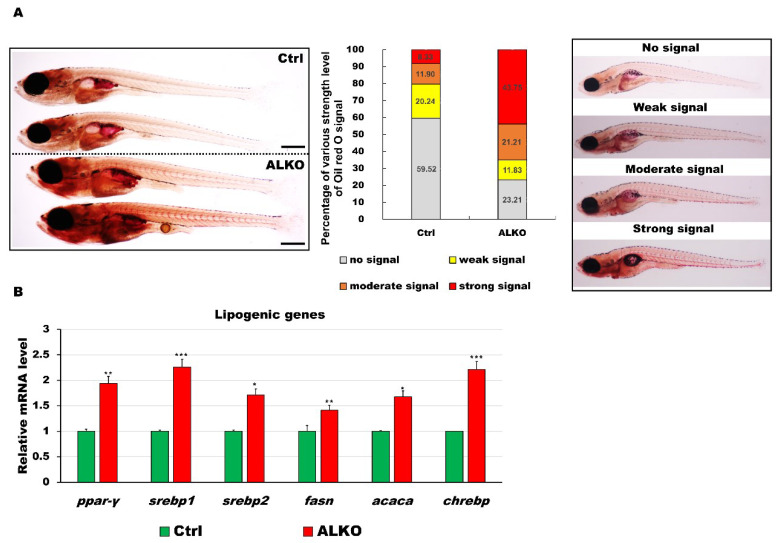
Defective Atgl led to energy imbalance and hyperlipidemia/hyperglycemia in ALKO larvae/adults. (**A**) Whole-mount Oil Red O staining of control (Heterozygous Tg (*Cmlc2-GFP-U6-gRNA^atgl^-4X*)) and ALKO at 21 dpf. Scale bar: 100 μm. Percentages of control and ALKO larvae with no, weak, moderate, and strong levels of lipidemia at 21 dpf were presented in the middle panel. (**B**) Molecule analysis of ALKO at 45 dpf represented an upregulation of the lipogenic genes *peroxisome proliferator-activated receptor gamma* (*ppar-γ)*, *sterol regulatory element binding transcription factor 1 (srebp1)*, *sterol regulatory element binding transcription factor 2 (srebp2)*, *fatty acid synthase (fasn)*, *acetyl-CoA carboxylase alpha (acaca)*, and *carbohydrate-responsive element-binding protein (chrebp).* Values of *ppar-y* = 1.9410 ± 0.0608, *srebp1* = 2.2597 ± 0.0562, *srebp2* = 1.7098 ± 0.0290, *fasn* = 1.4120 ± 0.0356, *acaca* = 1.6749 ± 0.0640, and *chrebp* = 2.2168 ± 0.1528 in ALKO group. Statistically significant differences from the controls are indicated by * *p* < 0.05, ** *p* < 0.01, and *** *p* < 0.001.

Several studies have demonstrated a lack of ATGL-attenuated hyperlipidemia/atherosclerosis [8], in vitro [40], in vivo [41,42], or in human patients with metabolic syndrome [19]. To validate the biological function of Atgl in zebrafish, we analyzed blood lipid and glucose profiles in our ALKO model. To examine the deposition of neutral lipids in ALKO, 21 dpf larvae were stained with Oil Red O (Figure 2A). The ALKO showed a strong signal in the dorsal aorta and caudal vein and a moderate signal in the visceral fat (Figure 2A, left). Notably, the incidence of solid signals in the ALKO group was significantly higher than the signals in the control group (Figure 2A, right), indicating that the absence of Atgl led to an early onset of hyperlipidemia in zebrafish. De novo lipogenesis was validated by checking the expression of lipogenic genes. All six selected lipogenic genes were elevated in the ALKO (Figure 2B), suggesting that the consequence of hyperlipidemia was attributed to positive feedback in lipogenesis. 

### 2.3. ALKO Adults Developed Metabolic Syndrome

**Figure 3:**
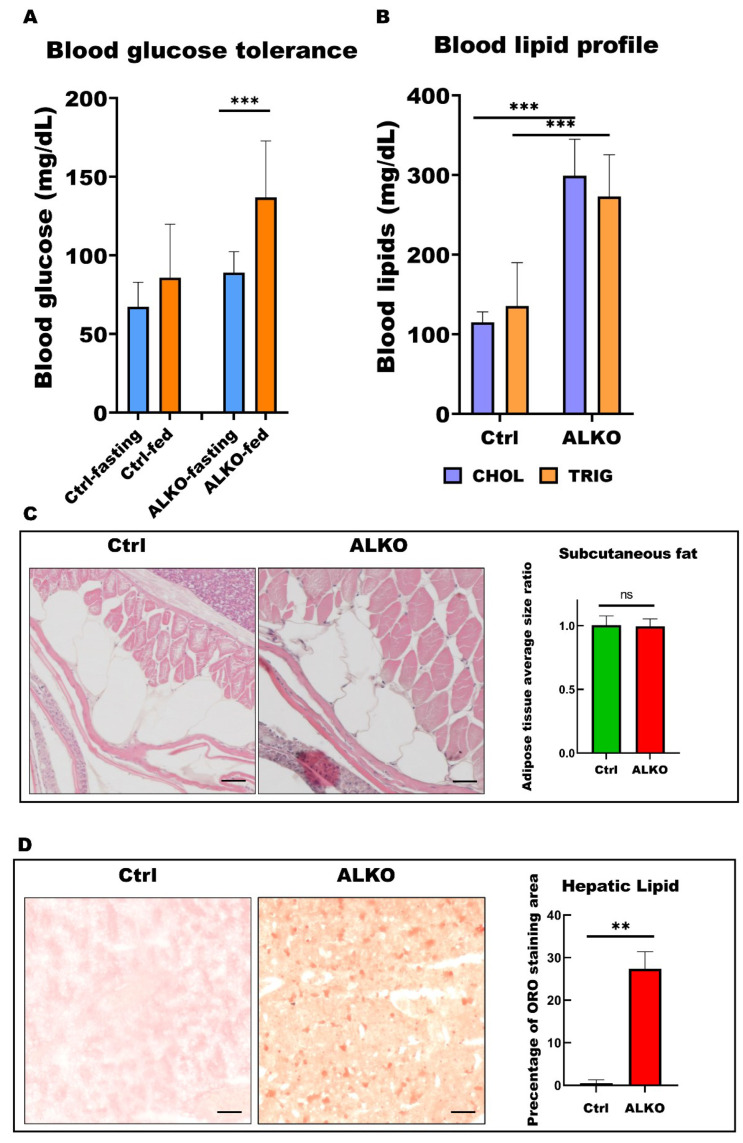
ALKO presented a typical metabolic syndrome outcome in adult. (**A**) Blood glucose contents of control and ALKO adults 5-month post-fertilization (mpf) at fasting and 90 mins after feeding. Values of Ctrl-fasting = 67.3636 ± 15.4872, Ctrl-fed = 85.75 ± 34.0619, ALKO-fasting = 89 ± 13.2581, and ALKO-fed = 136.833 ± 35.9415. (**B**) Blood lipid contents of control and ALKO adults at 5 mpf. CHOL: Total cholesterol content (mg/dL); TRIG: Total triglyceride content (mg/dL). Values of CHOL in Ctrl = 115 ± 13.2288, and ALKO = 299 ± 45.9239. Values of TRIG in Ctrl = 135.667 ± 54.3722, and AKOL = 273 ± 52.5071. (**C**) Characterization of subcutaneous fat in ALKO and control at 5 mpf. Scale bar: 50 μm. Values of adipose tissue average size ratio in Ctrl = 1.0041 ± 0.0738, and ALKO = 0.9946 ± 0.0595. (**D**) Histological images of hepatic ORO sections revealed a non-alcohol fatty liver phenotype in ALKO at 5 mpf. Scale bar: 50 μm. Percentage of ORO staining area in Ctrl = 0.481 ± 0.7925, and ALKO = 27.356 ± 4.0251. Statistically significant differences from the controls are indicated by ** *p* < 0.01, and *** *p* < 0.001.

Several previous studies have indicated that ATGL is also essential for insulin secretion [43,44]. Thus, we also measured the blood glucose level in adult ALKO. Blood analysis revealed a higher level (1.25-fold) of glucose content and worse insulin tolerance at 90 min after feeding in ALKO (Figure 3A). The blood lipid profiles were evaluated in the ALKO with a significant increase in cholesterol and triglyceride contents (Figure 3B), consistent with the early-stage observation (Figure 3B). Although the histology of subcutaneous fat shares the similar pattern in ALKO and control (Figure 3C), plenty of hepatic lipid accumulation served later outcome of metabolic disturbance occurred in ALKO only (Figure 3D).

### 2.4. Defective Atgl Enlarged the Ventricle Size in Zebrafish

**Figure 4:**
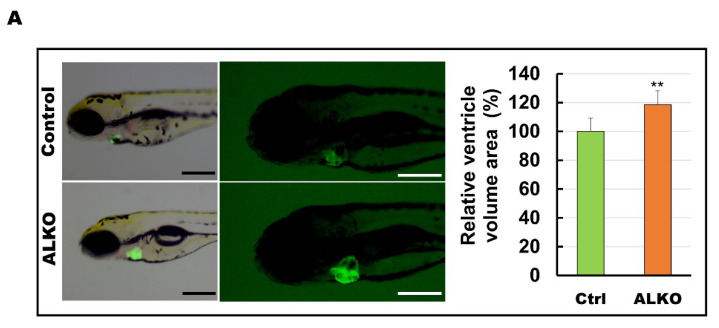

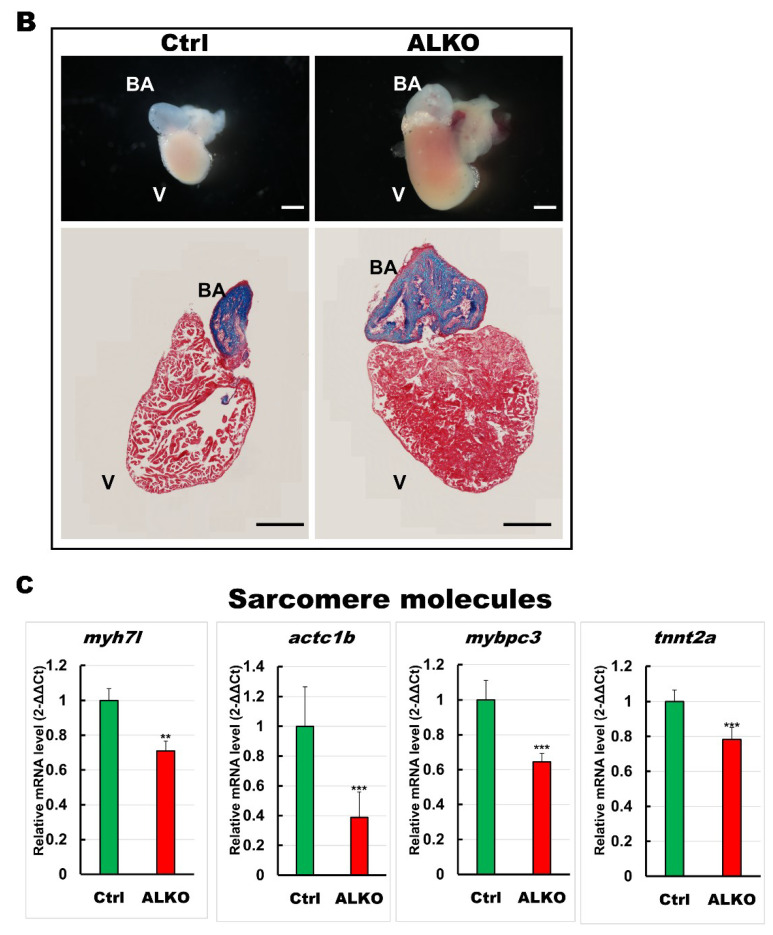
Hypertrophic hearts are evident in ALKO zebrafish in the larvae/adult stage. (**A**) Defective Atgl enlarged the ventricle size in ALKO at 5 dpf. Scale bar: 500 μm. The relative ventricle area was measured by the ventricle area of the control and ALKO 5 dpf embryo. Percentage of ventricle volume area in Ctrl = 100 ± 8.0138, and ALKO = 118.6974 ± 12.1794. (Control, *n* = 9; ALKO, *n* = 11). (**B**) Upper: The later view of the hearts in control and ALKO at 5 mpf. Lower: Paraffin-embedded sections of the hearts of control and ALKO at 5 mpf with Masson trichrome stained. Scale bar: 200 μm. B.A.: bulbus arteriosus, V: ventricle. (**C**) Molecule analysis of ALKO demonstrated the downregulation of sarcomere genes in the ventricle of 5 mpf ALKO (*n* = 3). Values of *myh7l* = 0.7088 ± 0.0564, *actc1b* = 0.3882 ± 0.145, *mybpc3* = 0.6449 ± 0.0474, and *tnnt2a* = 0.7824 ± 0.0671 in ALKO group. Statistically significant differences from the controls are indicated by ** *p* < 0.01, and *** *p* < 0.001.

Depletion of ATGL interferes with the metabolic state and contributes to cardiomyopathy [17,45]. We hypothesized that Atgl might play a vital role in myocardial protection in our zebrafish model. Hence, we next examined whether Atgl deficiency altered the size of the ventricle in ALKO. The ventricular volumes of ALKO were slightly increased (1.2-fold) compared to those of the control group (Figure 4A). The histological sections and lateral views of the hearts of ALKO zebrafish revealed a typical phenomenon of cardiac hypertrophy compared to the wild type (Figure 4B). As expected, the expression levels of sarcomere molecules including *myosin heavy chain 7-like* (*myh7l), actin alpha cardiac muscle 1b (actc1b), myosin binding protein C3 (mybpc3),* and *troponin T type 2a (tnnt2a)*, which served as biomarkers of myocardial function, decreased significantly (Figure 4C). Taken together, these results indicate that Atgl deficiency leads to a dramatic elevation of ventricular volume in zebrafish. 

### 2.5. Hypertrophic Heart in ALKO Adults due to Activated Immune Response

**Figure 5:**
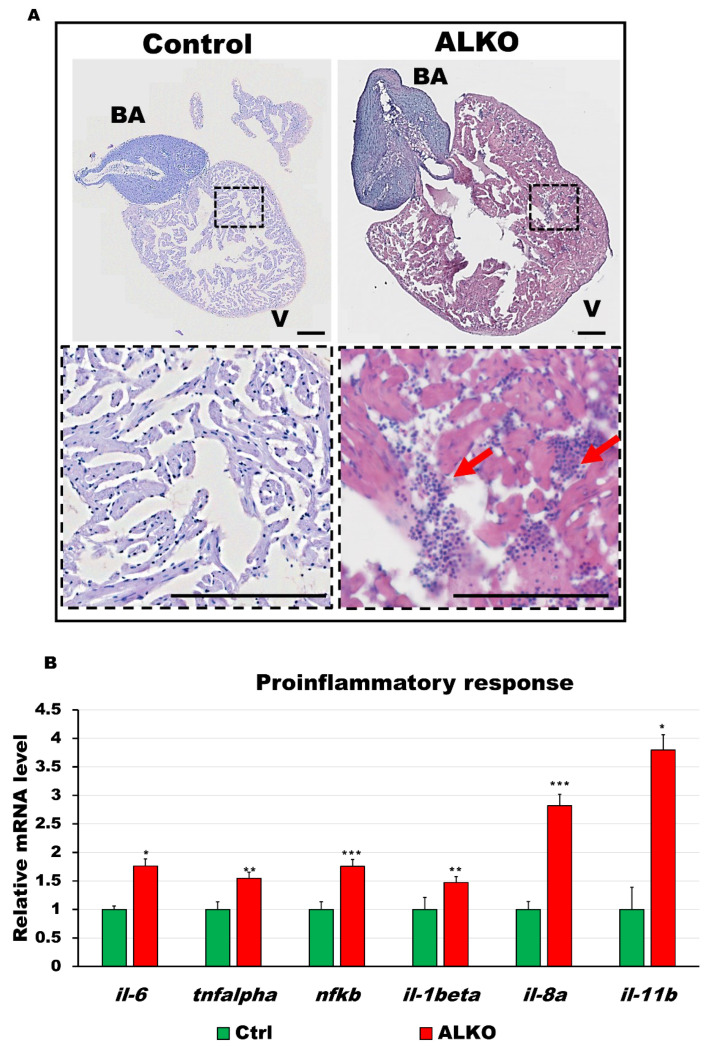
Depletion of Atgl activated immune response in the ventricle of ALKO. (**A**) Hematoxylin and eosin-stained sections of whole hearts of control and ALKO at 5 mpf. Scale bar: 100 μm. B.A.: bulbus arteriosus, V: ventricle. (**B**) Upregulated proinflammatory markers of ALKO ventricles revealed by RT-qPCR. In the ALKO group, *il6* = 1.7615 ± 0.0834, *tnfalpha* = 1.5474 ± 0.2950, *nfkb* = 1.7564 ± 0.4665, *il-1beta* = 1.4744 ± 0.0689, and *il-11b* = 3.7973 ± 0.7233. Statistically significant differences from the controls are indicated by * *p* < 0.05, ** *p* < 0.01, and *** *p* < 0.001.

To examine the link between metabolic syndrome and hypertrophic heart in ALKO, we next focused on the immune response involved. Inflammatory signals such as interleukin (IL)-1, IL-6, IL-8, tumor necrosis factor-alpha (TNF-α, and nuclear factor-kappa B (NF-kb) are usually elevated by metabolites under morbidly obese conditions [46]. To provide direct evidence, the whole hearts of ALKO adults were sectioned and imaged. Besides the increased size of the ventricle that was shown in Figure 3, immune infiltration was also observed only in the hearts of ALKO (Figure 5A, the red arrows indicate immune-infiltrated regions in the zoom-in areas of heart sections). The elevated inflammatory response in the myocardial tissue of ALKO was examined by checking proinflammatory gene signals; significant increases in the activity of *il-6, il-1β, il-8a, il-11b, tnf-α,* and *nf-kb* were observed (Figure 5B). These results suggest that the activation of the immune response stimulates cardiac remodeling.

### 2.6. Atgl Ablation Led Myocardial Remodeling Contributed to Cardiac Dysfunction in Zebrafish

**Figure 6:**
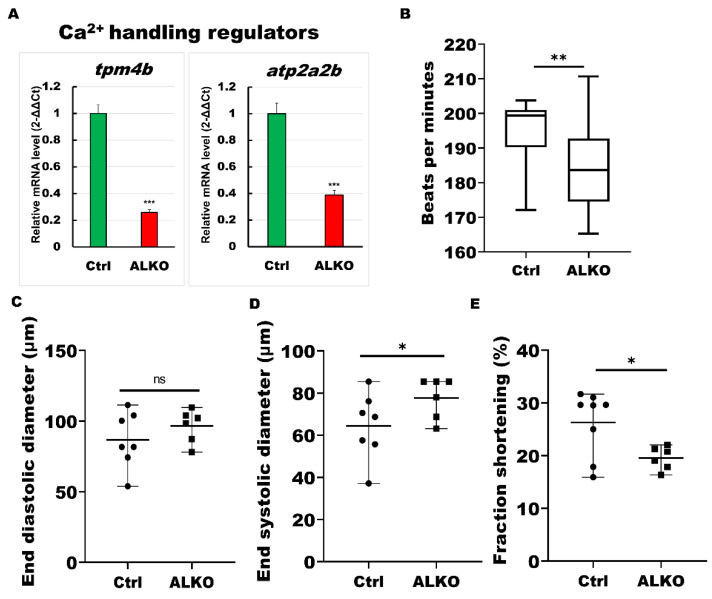
ALKO developed a dysfunctional heart. (**A**) RT-qPCR of ALKO ventricles at 5 mpf represented downregulation of calcium handling regulators: *tropomyosin 4-2* (*tpm4b*) and *ATPase sarcoplasmic/endoplasmic reticulum Ca^2+^ transporting 2b* (*atp2a2b*). In ALKO group, values of *tpm4b* = 0.258 ± 0.0201 and *atp2a2b* = 0.3882 ± 0.0341. (**B**) Heart rate assay revealing ventricle contraction. The heart rhythm analysis displayed impaired cardiac function in the ALKO compared to the control group at 120 hpf. Values of Ctrl = 194.538 ± 11.5407 and ALKO = 184.304 ± 13.0617. (**C**) End diastolic diameter showed no significant difference in ALKO group. Values of Ctrl = 86.7763 ± 19.9123 and ALKO = 96.595 ± 11.7485. (**D**) End systolic diameter increased in 120 hpf ALKO larvae. Values of Ctrl = 64.4851 ± 15.8247 and ALKO = 77.7095 ± 9.718. (**E**) The fraction shortening assay showed that the ALKO group exhibited a significant decrease compared to the control group. The fraction shortening, calculated as end-diastolic diameter (EDD) and end-systolic diameter (ESD), was measured in % as FS% = (EDD-ESD)/(EDD) × 100%. Percentage of Ctrl = 26.284 ± 6.1512 and ALKO = 19.5556 ± 2.185. Statistically significant differences from the controls are indicated by * *p* < 0.05, ** *p* < 0.01, and *** *p* < 0.001.

A significant reduction in sarcomere molecules was observed in the ventricles of ALKO, indicating cellular cardiac remodeling. We then evaluated the calcium regulators that support motor function in cardiac contractility and serve as our model’s downstream molecules of sarcomere signals. As expected, Ca^2+^ signaling pathways were downregulated in the ventricular tissue of ALKO adults (Figure 6A). We next analyzed the heart morphology, including heart rhythm (Figure 6B), end diastolic diameter (Figure 6C), end systolic diameter (Figure 6D), and fraction shortening (Figure 6E) of ALKO via an ImageJ software-based assay. All cardiac functional markers except end diastolic diameter presented lower values than the control group, revealing reduced heart contractility in ALKO zebrafish.

## 3. Discussion

Blockade of lipolysis usually results in metabolic syndrome-related symptoms [47]. ATGL mediates energy homeostasis globally. Thus, unsurprisingly, inactivated ATGL is highly relevant to diseases in mice and humans [48,49,50]. Human patients with ATGL mutations have progressive cardiomyopathy and triglyceride accumulation, which are characterized by multiple tissues [51,52]. Previous genetic mouse models displayed loss or gain of functional ATGL in a cellular or tissue manner [53]. Mouse models and clinical evidence have advanced our knowledge of the physiological role of ATGL in lipolysis and energy metabolism in adipose tissue and the pathological effects of ATGL deficiency in mice and humans. However, whether basal stimulated cardiomyopathy is caused by ATGL-mediated lipolysis remains unclear.

Numerous studies have demonstrated that transverse aortic constriction [54], diabetes [55], and obesity [56] do not cause cardiac dysfunction once cardiomyocyte-specific ATGL overexpression chronically reduces triglyceride accumulation in the heart. Chronically augmented ATGL exacerbated lipolysis in cardiac tissue, reducing fatty acids and increasing glucose oxidation. ATGL expression is triggered in the heart in response to diabetes or obesity, suggesting an adaptive but inadequate response to pathological increases in cardiac triglyceride [55,56]. Zhang et al. reported that heart-specific overexpression of ATGL attenuates lipid accumulation in the heart in a mouse model [56,57]. Consistent with these previous studies, the outcomes of typical metabolic symptoms were also observed in our ALKO mutant with hyperlipidemia (Figure 2A and Figure 3B), hyperglycemia (Figure 3A), and slight non-alcoholic fatty liver disease (Figure 2A and Figure 3D). The elevated lipogenic signals suggested that a positive signal loop occurred in the ALKO mutant owing to immune activation [58] and hyperglycemia [59]. Interestingly, depletion of the cofactor of ATGL, CGI-58 attenuated atherosclerotic lesion formation and showed a similar pattern to ATGL depletion in mice fed a high-fat or high-cholesterol diet [42]. 

**Figure 7:**
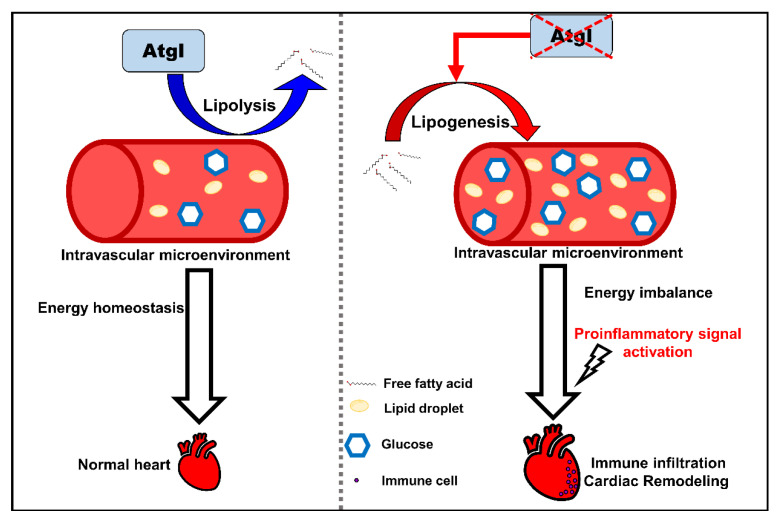
The schematic diagram summarizes the findings in this study. Defective Atgl activated lipogenic pathway leads to an energy imbalance microenvironment in the circulation system. Excess metabolites enhance cardiac remodeling by activating proinflammatory signals and immune cell recruitment.

The development of lipotoxic CM is strongly linked to immune infiltration by inflammatory cytokines, such as transforming growth factor-beta and IL-6 [9,10]. Genetic myocarditis, an indicative DCM symptom, is a late-stage consequence of chronic inflammation [60,61]. Severe myocarditis was observed in our ALKO adults (Figure 5A) and presented a similar pattern to murine heart sections by hematoxylin and eosin (H&E) staining [62]. Notably, the thinner ventricle wall of ALKO compared to wild-type adults (Figure 5A) and larvae (data not shown) indicated the typical DCM phenomenon in zebrafish [63]. Kamel et al. generated a zebrafish *tnnt2a* mutant, TnT-RK94del, for genetic CMs. Larvae carrying the TnT RK94del mutation Ca^2+^ disrupt calcium dynamics associated with increased sensitivity, resulting in diastolic dysfunction. Adult zebrafish carrying the heterozygous TnT-RK94del mutation displayed CM, as observed in patients with TnT mutations [27]. Consistent with our study, the inactivation of sarcomere signals (Figure 4C), Ca^2+^ regulators (Figure 6A), and loss of cardiac contractility (Figure 6D,E) demonstrate that loss of Atgl leads to cardiac remodeling via regulated energy metabolic imbalance, proinflammatory cytokine activation, and cardiac contractility (Figure 7).

The difference between tissue-specific KO and global KO of ATGL has been previously examined in several murine studies [53]. Local defective ATGL may clear lipid metabolism in the target tissue without synergistic effects. Acute pharmacological suppression of ATGL and adipocyte-specific gene deletion delay the development of heart failure [64]. However, in sharp contrast to ATGL depletion in cardiomyocytes, this results in a severe cardiac phenotype. Haemmerle et al. reported that ATGL deficiency in mice results in excessive lipid accumulation, heart failure, and fatal cardiomyopathy [16]. Han et al. reported that ATGL KO contributes to cardiac dysfunction and poor remodeling, possibly connected to the proteasome-phosphatase and tensin homolog-mammalian target of rapamycin-autophagy pathway [65]. However, the global KO of ATGL provides the whole picture of lipid metabolism crosstalk rather than the pharmacological treatment of ATGL inhibition [66]. Takahara et al. demonstrated that manipulating ATGL improves cardiac dysfunction in a mouse model of left ventricular pressure overload [67]. Thiele et al. reported the cardioprotective effects of Atglistatin, a pharmacological inhibitor targeting ATGL mainly in adipose tissue, against catecholamine-induced cardiac injury [64]. In this regard, we demonstrated an obesity-induced CM model in a defective genetic environment in zebrafish via Atgl ablation. We believe this zebrafish model with genetically deficient Atgl will provide a better opportunity to discover therapeutic approaches for human patients [21,52,68].

In summary, we demonstrated that Atgl plays a vital role in the development of cardiomyopathy via lipid/glucose metabolic regulation, myocardial immune initiation, and cardiac remodeling. We characterized the role of Atgl from lipid metabolism regulation linkage to inflammatory initiation and cardiac morphology changes. Thus, our findings may help to elucidate the underlying mechanism and pharmacy-approach discovery for patients with obesity-related CM.

## 4. Materials and Methods

### 4.1. Generation and Maintenance of Atgl Mutant Zebrafish

The transgenic zebrafish lines, Tg (*-2.5β-Act:Cas9-2A-GFP*), which show global expression of GFP and Cas9 driven by the β-action promoter, and gRNAs*^atgl^* [Tg (*-0.3 cmlc2:GFP-U6-gRNA^atgl^-4X*)], which offers four global selective *atgl* gRNAs (listed in Supplemental Table S1) expression driven by the U6 promoter, were maintained in a stable condition with a 14/10-h light-dark cycle at 28 °C. All animal studies were approved by the Institutional Animal Care and Use Committee guidelines.

### 4.2. In Situ Hybridization 

In situ hybridization was performed as previously described [69]. Using the primers listed in Table 1, the gene-specific probes were cloned by PCR into a pGEM-T Easy TA cloning vector (Promega). In vitro transcription was used to create antisense probes using the DIG RNA Labeling Kit (SP6/T7) (Roche Applied Science, Penzberg, Germany).

### 4.3. Whole-Mount Oil Red O Staining

All samples of zebrafish larvae were treated with 4% paraformaldehyde solution in PBS fixed in 1.5 mL Eppendorf tubes overnight at 4 °C. Each group of control and mutant larvae was rinsed three times with PBS before staining. Sixty percent isopropyl alcohol was used to pre-stain each group of larvae for at least 30 min. Freshly prepared 0.6% Oil Red O solution was added for staining and incubated at 4 °C for 1 h. The staining process was stopped by washing twice with PBS. Finally, they were stored in 70% glycerol and imaged using a model Stemi 305 bright-field dissecting microscope (Carl Zeiss, Jena, Germany).

### 4.4. Blood Analysis

Blood samples were collected as previously described [70]. Each group of collected blood samples was pooled to a volume of at least 5 μL for the glycose test (Accu-chek; Roche, Basel, Switzerland) and 45 μL for the total triglyceride/cholesterol test (CardioChek, Beijing, China).

### 4.5. Histology

The dissected hearts were rinsed and fixed with Zinc Formal-Fixx (Thermo Fisher Scientific, Waltham, MA, USA). The fixed heart tissues were dehydrated with several degrees of ethanol and embedded in paraffin. H&E staining was performed, and Masson’s trichrome staining was performed using a Masson’s trichrome stain kit (Polysciences, Inc., Warrington, PA, USA). Oil red O staining was performed as previously described [71]. 

### 4.6. RT-qPCR

Total RNA extraction was performed using TRIzol reagent (Invitrogen, Carlsbad, CA, USA) or the Total RNA Mini Kit (Bioman, Taipei, Taiwan) and reverse transcribed using the first-strand cDNA synthesis kit (Fermentas, Waltham, MA, USA). Real-time RT-PCR was performed using a PerfeCTa SYBR Green FastMix Real-Time PCR System (Quantabio, Beverly, MA, USA), and the final data were derived from 2−ΔΔCt, and fold-induction was computed using the Ct method as follows: ΔΔCt = (CtTarget − CtHousekeeping) ALKO − (CtTarget − CtHousekeeping) Control. GenBank accession numbers of the selected genes and primer sequences are listed in Supplemental Table S2.

### 4.7. Morphology Analysis of Heart Function

Heart rate analysis was performed as previously described [72]. The videos were recorded using a digital charge-coupled device (LeadView 2000AIO PLUS and 8000 AIO LITE, Leader, Taipei, Taiwan) at a speed of 60 frames per second with full high-definition resolution. ImageJ software was used to calculate the heart rate in beats per minute, end-diastolic diameter, and end-systolic diameter. Fraction shortening, calculated as end-diastolic diameter (EDD) versus end-systolic diameter (ESD), was measured as FS% = (EDD-ESD)/(EDD) × 100%, as previously described [24].

### 4.8. Statistical Analysis

All values presented in each graph are mean ± standard error of the mean. Statistical analysis was performed using analysis of variance (ANOVA) followed by Bonferroni post-tests using GraphPad Prism 9.0 software (GraphPad, San Diego, CA, USA). Statistical significance was set at *p* < 0.05.

## Figures and Tables

**Table 1:** Primer sequences used for in-situ hybridization probes.

Gene	Accession	Forward Primer	Reverse Primer
*atgl*	XM_005174256	CTGTTCCCACCTGATCCACT	TGACGTCCAGCATTGAAGAG

## Data Availability

Not applicable.

## Supplementary Materials

The following supporting information can be downloaded at https://www.mdpi.com/article/10.3390/ijms24010117/s1, Table S1: gRNA sequences used for the *atgl*-4X gRNA cassettes. Table S2: Primer sequences used for quantitative RT-PCR.

## Abbreviations

ATGL	Adipose triglyceride lipase
ALKO	Atgl knockout
CM	Cardiomyopathy
gRNAs	guide RNAs
PGC1	peroxisome proliferator-activated receptor gamma cofactor 1
DCM	dilated cardiomyopathy
TTNtv	titins
Cmlc2	cardiac myosin light chain 2
Indel	Insertion/deletion
dpf	days post-fertilization
hpf	h post-fertilization
mpf	month post- fertilization
ppar-γ	peroxisome proliferator-activated receptor gamma
srebp	sterol regulatory element binding transcription factor
fasn	fatty acid synthase
acaca	acetyl-CoA carboxylase alpha
chrebp	carbohydrate-responsive element-binding protein
myh7l	myosin heavy chain 7-like
actc1b	actin alpha cardiac muscle 1b
mybpc3	myosin binding protein C3
tnnt2a	troponin T type 2a
il	interleukin
tnf-α	tumor necrosis factor-alpha
nf-kb	nuclear factor-kappa B
tpm4b	tropomyosin 4-2
atp2a2b	ATPase sarcoplasmic/endoplasmic reticulum Ca^2+^ transporting 2b
EDD	end-diastolic diameter
ESD	end-systolic diameter
FS	fraction shortening
H&E	hematoxylin and eosin
ANOVA	analysis of variance
