# Supplementary material for: Deficiency of Adipose Triglyceride Lipase Induces Metabolic Syndrome and Cardiomyopathy in Zebrafish
Source: Int J Mol Sci. 2022 Dec 21;24(1):117. doi: 10.3390/ijms24010117 (PMC9820674; doi:10.3390/ijms24010117)
Supplement: Supplementary file 1 [file ijms-24-00117-s001.zip › ijms-2073325-supplementary.pdf]

Supplementary Table S1. gRNA sequences used for the *atgl*-4X gRNA cassettes

Sequence Names	gRNAs
atgl-gRNA1	CCGTGGAATATTTCGTTCGCAGG
atgl-gRNA2	CGGAGTGGCCAGCTGTCTACAGG
atgl-gRNA3	CTGGTGTCGCGGTCTTCGAGAGG
atgl-gRNA4	CAGCCTGACTCCTTAAACACAGG

*PAM sites (Cas9 protein binding sites) labeled in red.

Supplementary Table S2. Primer sequences used for quantitative RT-PCR

Gene	Accession	Forward primer	Reverse primer
atgl	XM_005174256	TCATATTTACGGCGCGTCC	CGCTTTCGGGCTTCTTTAG
srebp1	NM_001105129	AAAGCCATCGAGTACATCCG	CACCAGATCCTTCAGAGACTTG
srebp2	NM_001089466	GAGATAAAGCGGACCCCATC	CAGAAACTCCAGAACCCCAG
pparγ	NM_131467.1	GGAGAACACATACAGAGCACAG	ACGGATCTTCAGGAGTTTTGG
fasn	XM_021472581.1	TGGCAAAGAGTCTGAAGCTG	AGATCAACAGCCTTGTCCAC
acaca	NM_001271308.1	CTGTGAAAGCGTATTTGTGGG	TGCGGATCTGTTTGAGGATG
chrebp	XM_021476328	GAATCTCAGGTCAAGGGTCAC	TCTGCGACTCTGACTGTTTTC
il-6	NM_001261449.1	CAGAGACGAGCAGTTTGAGAG	AGACATCTTTCCGTGCTGAAG
tnfa	NM_212859	GCTTATGAGCCATGCAGTGA	TGCCCAGTCTGTCTCCTTCT
nf-κb	NM_001001840.3	ATGACGAGCCTCTTTTCCTG	TCTCTTCCGTGGGTTAAACAC
il-11b	XM_021468285	ACGGAATAGTGACGAATTGGG	CAATGTGGGATGGACGATCTC
il-1b	AY340959.1	TCAAACCCCAATCCACAGAG	TCACTTCACGCTCTTGGATG
il-8a	XM_009306855.3	CATTGAAACAGAAAGCCGACG	GATCTCCTGTCCAGTTGTCATC
myh7l	NM_001077464.2	GGATTGTACCAGAAGTCCTCTC	ATTCTCCCTGTGAAGTGCTG
actc1b	NM_131591.1	AGGCTACTCTTTCGTGACAAC	CGAAGTCCAGAGCCACATAG
mybpc3	NM_001044349.2	GAAAACTCAGCACCAGAAAGC	TTCACCAACATTCACCTCCC
tnnt2a	NM_152893	TGATCGACGCAAACCTCTG	TGCCGAACTGATACTGTAACTC
tpm4b	NM_199675.2	ATATGAAGAGGTTGCCCGTAAG	GCTTCCAAAGACTTGAGGTTG
atp2a2b	NM_001030277.1	AACGCTATTGTAGGTGTCTGG	CCTGGCTTTGATCCTCTGTAC
